# Relaxation mode analysis for molecular dynamics simulations of proteins

**DOI:** 10.1007/s12551-018-0406-7

**Published:** 2018-03-15

**Authors:** Ayori Mitsutake, Hiroshi Takano

**Affiliations:** Department of Physics, Faculty of Science and Technology, Keio University, Tokyo, Japan

**Keywords:** Protein, Simulation, Analysis, Dynamics

## Abstract

Molecular dynamics simulation is a powerful method for investigating the structural stability, dynamics, and function of biopolymers at the atomic level. In recent years, it has become possible to perform simulations on time scales of the order of milliseconds using special hardware. However, it is necessary to derive the important factors contributing to structural change or function from the complicated movements of biopolymers obtained from long simulations. Although some analysis methods for protein systems have been developed using increasing simulation times, many of these methods are static in nature (i.e., no information on time). In recent years, dynamic analysis methods have been developed, such as the Markov state model and relaxation mode analysis (RMA), which was introduced based on spin and homopolymer systems. The RMA method approximately extracts slow relaxation modes and rates from trajectories and decomposes the structural fluctuations into slow relaxation modes, which characterize the slow relaxation dynamics of the system. Recently, this method has been applied to biomolecular systems. In this article, we review RMA and its improved versions for protein systems.

## Introduction

Molecular dynamics simulation is widely used for protein research. In general, the focus of this research is to extract information on the physical properties of individual proteins. The results from such simulations are then often compared with experimental results. Since these experiments are generally conducted in solvents, it is necessary to simulate protein and water molecular systems, which are complicated systems. These simulations are conducted for a variety of purposes such as to analyze the stability and dynamics of the structures around crystal structures and to determine folding from an extended structure into a native structure. There are three difficulties in current approaches for protein simulations (Freddolino et al. 2010). The first is the potential function of the protein systems. In recent years, it has become possible to evaluate the molecular force field by improving the sampling, and accuracy has consequently improved. The second problem is related to the sampling. With respect to the folding mechanism, simulation at the millisecond scale is necessary. Recently, it has become possible to perform simulations at the millisecond scale by using special hardware such as Anton (Lindorff-Larsen et al. 2011, 2012; Dror et al. 2012, Lane et al. 2013), but sampling problems still exist for complex systems such as ligand-binding systems and other even more complex systems. The third issue is related to the analysis methods. It is important to extract the characteristic degrees of freedom (order parameters) from the complex protein movements obtained from simulations, which are good indicators for analyzing trajectories.

In normal mode analysis, the normal mode near the minimum point of the potential energy of the protein molecule is obtained (Go et al. 1983; Brooks and Karplus 1983; Levitt et al. 1985). Langevin mode analysis investigates modes around the native structure, including the water effect (Lamm and Szabo 1986; Kottalam and Case 1990; Kitao et al. 1991; Hayward et al. 1993). An elastic network model and Gaussian network model approximately estimate normal modes with large amplitudes by using the harmonic potential of coarse-grained models (Tirion 1996; Baher et al. 1997; Tama and Sanejouand 2001; Cui and Bahar 2005; Miyashita and Tama 2008). This method extracts collective modes with large amplitudes in the case of huge protein systems such as viruses, because huge proteins have rigid-like motions (Tama and Brooks III 2002).

Principal component analysis (PCA), also called quasiharmonic analysis or the essential dynamics method (Levy et al. 1984; Ichiye and Karplus 1991; Abagyan and Argos 1992; Garcia 1992; Hayward et al. 1993; Amadei et al. 1993; Kitao and Go 1999), is one of the most popular methods adopted for analyzing the structural fluctuations around the average structure. The modes with large structure fluctuations are extracted and are regarded as cooperative movement, and the relation of these fluctuations with function has been widely investigated. The obtained modes are also used as the axis of the free-energy surface. Moreover, various other analysis methods have been proposed, such as full correlation analysis (Lange and Grubmüller 2007), subspace joint approximate diagonalization of eigenmatrices (Sakuraba et al. 2010), and wavelet analysis (Kamada et al. 2011), among others (Moritsugu et al. 2015; Matsunaga et al. 2015).

In recent years, it has become possible to perform an extensively long simulation; thus, development of dynamic analysis methods to identify the local minimum-energy states and analyze the transitions between them is required. Accordingly, many methods to analyze the dynamics and kinetics of protein simulations have been developed (Zuckerman 2010; Komatsuzaki et al. 2011; Bowman et al. 2014). In particular, the Markov state model has been presented and applied to many protein systems (Schütte et al. 1999; Swope et al. 2004; Singhal et al. 2004; Chodera et al. 2006, 2007; Chodera and Noé 2014; Noé et al. 2007; Noé and Fischer 2008; Noé and Clementi 2017 Buchete and Hummer 2008; Prinz et al. 2011; Pérez-Hernández et al. 2013; Schwantes and Pande 2013; Schwantes et al. 2014; Bowman et al. 2014; Wu et al. 2017). The Markov state model can analyze transitions between local minimum-energy states, which are identified from clustering analysis methods. This is a powerful method for analyzing dynamics in the context of both long and short simulations of proteins.

Relaxation mode analysis (RMA) was developed to investigate the “dynamic” properties of spin systems (Takano and Miyashita 1995) and homopolymer systems for Monte Carlo (Koseki et al. 1997) and molecular dynamics (Hirao et al. 1997) analyses, and has been applied to various polymer systems (Hagita and Takano 2002; Saka and Takano 2008; Iwaoka et al. 2015; Natori and Takano 2017) to investigate their slow relaxation dynamics (de Gennes 1984; Doi and Edwards 1986). Recently, RMA has also been applied to biomolecular systems (Mitsutake et al. 2011; Mitsutake et al. 2005; Mitsutake and Takano 2015; Nagai et al. 2009, 2013). RMA approximately estimates slow relaxation modes and rates from trajectories obtained from simulations.

The relaxation modes {*X*_*p*_} satisfy

1 $$ \langle X_{p}(t)X_{q}(0) \rangle=\delta_{p,q} e^{-\lambda_{p} t}.  $$

Here, 〈*A*(*t*)*B*(0)〉 denotes the equilibrium correlation of *A* at time *t* and *B* at time 0:

2 $$ \langle A(t)B(0) \rangle =\sum\limits_{Q,Q^{\prime}}A(Q)T_{t}(Q|Q^{\prime})B(Q^{\prime})P_{\text{eq}}(Q^{\prime}) ,  $$

where *T*_*t*_(*Q*|*Q*^′^) is the conditional probability that the system is in state *Q* at time *t* given that it is in state *Q*^′^ at time *t* = 0. Further, *P*_eq_(*Q*^′^) denotes the probability that the system is in state *Q*^′^ at equilibrium. The relaxation rate of *X*_*p*_ is denoted by *λ*_*p*_. The relaxation time is given by 1/*λ*_*p*_. Note that the relaxation modes and rates are given as left eigenfunctions and eigenvalues of the time evolution operator of the master equation of the system, respectively, from the viewpoint of the statistical mechanics (Hirao et al. 1997; Koseki et al. 1997; Mitsutake and Takano 2015) (see the “Relaxation modes {*X*_*p*_} and rates *λ*_*p*_” section). The point of RMA is that we consider the variational problem, which is equivalent to the eigenvalue problem of the time evolution operator, and choose an appropriate trial function to estimate the slow relaxation modes and rates in the system (see the “RMA” section). From these processes, we obtain the generalized eigenvalue problem of the time correlation matrices for two different times. From the eigenvectors and eigenvalues, we approximately estimate slow relaxation modes and rates.

Conventional RMA approximately estimates slow relaxation modes by solving the generalized eigenvalue problem of the time correlation matrices of coordinates for two different times, *C*(*τ* + *t*_0_) and *C*(*t*_0_), which are calculated from the trajectory. Recently, dynamical analysis methods for molecular simulations of biopolymer systems have been developed to investigate slow dynamics. In these techniques such as time structure-based independent component analysis (tICA) (Naritomi and Fuchigami 2011, 2013), time-lagged independent component analysis (TICA) (Pérez-Hernández et al. 2013; Schwantes and Pande 2013), and dynamic component analysis (DCA) (Mori et al. 2015, 2016), time correlation matrices of certain physical quantities or states are used. (Note that tICA is a special case of RMA with *t*_0_ = 0. See Mitsutake et al. (2011) and Naritomi and Fuchigami (2011) for more details on the differences between tICA and RMA.) In tICA, TICA, and DCA, the time correlation functions C(*τ*) and C(0) are used, whereas C(*τ* + *t*_0_) and C(*t*_0_) are used in RMA. The relaxation modes and rates are given as left eigenfunctions and eigenvalues of the time evolution operator of the master equation of the system, respectively. From this point of view, RMA is related to Markov state models. (The relationship among the Markov state model, tICA, and TICA is explained in Pérez-Hernández et al. (2013), Schwantes and Pande (2013), and Mitsutake and Takano (2015).) The combination method of tICA and a Markov state model was also proposed (Pérez-Hernández et al. 2013; Schwantes and Pande 2013). A Markov state model was constructed from clustering in the subspace determined by tICA.

In this review, we first provide a definition of relaxation modes and rates from the viewpoint of the statistical mechanics in the “Relaxation modes {*X*_*p*_} and rates *λ*_*p*_” section. The “RMA” section explains the original RMA (RMA with a single evolution time) and the process of RMA using coordinates for the trial function in detail. The “Improvement of RMA” section explains the improved versions of RMA, including RMA with multiple evolution times, principal component RMA (PCRMA), two-step RMA, and Markov-state RMA (MSRMA). Finally, in the “Application of RMA to a system with large conformational changes” section, we present results from studies in which RMA was applied to a system with large conformational changes. The “Conclusions” section provides conclusions and perspectives on the state of the field.

## Relaxation modes {*X*_*p*_} and rates *λ*_*p*_

In this section, we provide the definition of relaxation modes and rates from the viewpoint of the statistical mechanics (Risken 1989; Zwanzig 2001). The relaxation modes {*X*_*p*_} satisfy Eq. 1. The relaxation modes and rates are given as left eigenfunctions and eigenvalues of the time evolution operator of the master equation of the system, respectively. We first explain the relation in three types of simulations satisfying the detailed balance condition.

In a Monte Carlo simulation satisfying the detailed balance condition, the time evolution of the probability *P*(*Q*;*t*) that the biomolecule is in a state $Q= \left (\boldsymbol {r}_{1}^{\mathrm {T}},\boldsymbol {r}_{2}^{\mathrm {T}},\right .$ $\left .\cdots ,\boldsymbol {r}_{N}^{\mathrm {T}}\right )^{\mathrm {T}}$ at time *t* is described by a master equation:

3 $$ \frac{\partial}{\partial t}P(Q;t)= -\sum\limits_{Q^{\prime}} {\Gamma} (Q|Q^{\prime})P(Q^{\prime};t).  $$

Here, Γ(*Q*|*Q*^′^) denotes the (*Q*, *Q*^′^)-component of the time evolution matrix Γ, and ${\sum }_{Q^{\prime }}$ denotes the summation over all possible states. Γ(*Q*|*Q*^′^) is also chosen so that the detailed balance for the equilibrium distribution function *P*_eq_(*Q*) is satisfied:

4 $$ {\Gamma} (Q|Q^{\prime})P_{\text{eq}}(Q^{\prime}) ={\Gamma} (Q^{\prime}|Q)P_{\text{eq}}(Q).  $$

In the Brownian dynamics simulation, the time evolution of coordinates ***r***_*i*_,(*i* = 1,⋯ ,*N*) is given by the Langevin equation for a biomolecule with *N* atoms:

5 $$ \frac{d \boldsymbol{r}_{i}}{dt} = -\frac{1}{\zeta} \left[ -\frac{\partial}{\partial \boldsymbol{r}_{i}} U(\{ \boldsymbol{r}_{j} \})+\boldsymbol{w}_{i} \right] .  $$

Here, ***r***_*i*_(*t*) denotes the position of the *i* th atom at time *t*, and *ζ* is the friction constant. The interaction between atoms is described by the potential *U*({***r***_*i*_}) = *U*(***r***_1_,…,***r***_*N*_). The random force ***w***_*i*_(*t*) acting on the *i* th atom is a Gaussian white stochastic process and satisfies

6 $$ \langle w_{i,\alpha}(t) w_{j,\beta}(t) \rangle = 2 \zeta k_{B} T \delta_{\alpha,\beta}\delta_{i,j}\delta(t-t^{\prime}),  $$

where *w*_*i*, *α*_, *k*_*B*_, and *T* denote the *α*-component of ***w***_*i*_ (*α* = *x*, *y*, or *z*), the Boltzmann constant, and the temperature of the system, respectively. The Smoluchowski equation equivalent to Eq. 5 can be written as

7 $$ \begin{array}{ll} \frac{\partial}{\partial t}P(Q,t) & =-{\Gamma}(Q) P(Q,t) \\ & = \sum\limits_{i = 1} \frac{\partial}{\partial \boldsymbol{r}_{i}} \cdot \frac{1}{\zeta} \left\{ k_{B} T \frac{\partial}{\partial \boldsymbol{r}_{i}} + \frac{\partial U}{\partial \boldsymbol{r}_{i}} \right\} P. \end{array}  $$

Here, *Q* = {***r***_1_,…,***r***_*N*_} denotes a point in the phase space of the system, and *P*(*Q*, *t*)*dQ* denotes the probability that the system is found at time *t* in an infinitesimal volume *dQ* at point *Q* in the phase space. The time evolution operator Γ satisfies the detailed balance condition (Risken 1989):

8 $$ P_{\text{eq}}(Q^{\prime}){\Gamma}(Q) \delta(Q-Q^{\prime})= P_{\text{eq}}(Q){\Gamma}^{\dagger}(Q) \delta(Q-Q^{\prime}) ,  $$

where $P_{\text {eq}}(Q) \propto \displaystyle \exp \left [ -\frac {U (\{\boldsymbol {r}_{j}\} )}{k_{B}T} \right ]$. Here, Γ(*Q*)*δ*(*Q* − *Q*^′^) and the adjoint operator Γ^‡^(*Q*)*δ*(*Q* − *Q*^′^) act only on *Q* in *δ*(*Q* − *Q*^′^). In the matrix representation, so that Γ(*Q*)*δ*(*Q* − *Q*^′^) = Γ(*Q*|*Q*^′^) and Γ^‡^(*Q*)*δ*(*Q* − *Q*^′^) = Γ(*Q*^′^|*Q*), the detailed balance condition is the same as that in Eq. 4.

In a molecular dynamics simulation with the Langevin thermostat, the time evolution of coordinates ***r***_*i*_,(*i* = 1,⋯ ,*N*) is given by the Langevin equation for a biomolecule with *N* atoms:

9 $$ m_{i}\frac{d \boldsymbol{v}_{i}}{dt} = -\zeta \boldsymbol{v}_{i} -\frac{\partial}{\partial \boldsymbol{r}_{i}} U(\{ \boldsymbol{r}_{j} \})+\boldsymbol{w}_{i},  $$

with

10 $$ \frac{d \boldsymbol{r}_{i}}{dt}=\boldsymbol{v}_{i} .  $$

Here, ***r***_*i*_(*t*) and ***v***_*i*_(*t*) denote the position and the velocity of the *i* th atom at time *t*, respectively. The mass of the *i* th atom is denoted by *m*_*i*_ and *ζ* is the friction constant.

The Kramers equation, equivalent to Eqs. 9 and 10, can be written as

11 $$ \begin{array}{l} \frac{\partial}{\partial t}P(Q,t)=-{\Gamma}(Q) P(Q,t) \\ = \sum\limits_{i = 1}^{N} \left\{ \frac{\partial}{\partial \boldsymbol{r}_{i}} \cdot \boldsymbol{v}_{i} -\frac{1}{m_{i}}\frac{\partial}{\partial \boldsymbol{v}_{i}} \cdot \frac{\partial U}{\partial \boldsymbol{r}_{i}} - \frac{\zeta}{m_{i}}\frac{\partial}{\partial \boldsymbol{v}_{i}}\cdot \left( {\boldsymbol v}_{i} + \frac{k_{B} T}{m_{i}} \frac{\partial}{\partial \boldsymbol{v}_{i}} \right) \right\}P . \end{array}  $$

Here, *Q* = {***r***_1_,…,***r***_*N*_,***v***_1_,…,***v***_*N*_} denotes a point in the phase space of the system. The time evolution operator Γ satisfies the detailed balance condition:

12 $$ P_{\text{eq}}(Q^{\prime}){\Gamma}(Q) \delta(Q-Q^{\prime})= P_{\text{eq}}(\epsilon Q){\Gamma}^{\dagger}(\epsilon Q) \delta(\epsilon Q- \epsilon Q^{\prime}) ,  $$

where $P_{\text {eq}}(Q) \displaystyle \propto \exp \left (-\frac {1}{k_{B}T} \left [ \frac {1}{2} \sum\limits_{i} m_{i} \boldsymbol {v}_{i}^{2} + U (\{\boldsymbol {r}_{j}\} ) \right ] \right )$ and *P*_eq_(*Q*) = *P*_eq_(*𝜖Q*). Here, *𝜖Q* denotes the time-reversed state of the state *Q*, namely, *𝜖Q* = {*𝜖*_1_***r***_1_,…,*𝜖*_*N*_***r***_*N*_,.*𝜖*_*N*+ 1_***v***_1_,…,*𝜖*_2*N*_***v***_*N*_} with

13 $$ \epsilon_{i} = \left\{ \begin{array}{rcl} 1 & \text{ for } & i = 1,\ldots,N,\\ -1& \text{ for } & i=N + 1,\ldots,2N. \end{array} \right.  $$

In the matrix representation, the detailed balance condition is written as follows:

14 $$ {\Gamma}(Q|Q^{\prime})P_{\text{eq}}(Q^{\prime})={\Gamma}(\epsilon Q^{\prime}|\epsilon Q)P_{\text{eq}}(\epsilon Q).  $$

The time evolution equation of *P*(*Q*;*t*) of Eqs. 7 and 11 corresponds to Eq. 3 in the matrix representation. In Monte Carlo and Brownian dynamics, because only coordinates are the degrees of freedom in the system, *𝜖Q* = *Q*, the detailed balance condition in all three cases is given by Eq. 14.

We now consider the eigenvalue problem of the time evolution operator Γ(*Q*|*Q*^′^) of the master equation:

15 $$ \sum\limits_{Q} \phi_{n}(Q) {\Gamma}(Q|Q^{\prime})=\lambda_{n} \phi_{n}(Q^{\prime}).  $$

16 $$ \sum\limits_{Q^{\prime}} {\Gamma}(Q|Q^{\prime})\psi_{n}(Q^{\prime})=\lambda_{n} \psi_{n}(Q).  $$

Here, *ϕ*_*n*_(*Q*) and *ψ*_*n*_(*Q*) are the left and right eigenfunctions of the time evolution operator Γ with eigenvalue *λ*_*n*_, respectively. When we define a quantity $\hat {\phi }_{n}(Q)$ through

17 $$ \psi_{n}(Q)=\hat{\phi}_{n}(Q)P_{\text{eq}}(Q) ,  $$

then $\hat {\phi }_{n}(Q)=\phi _{n}(\epsilon Q)$. The eigenfunctions are chosen to satisfy the orthonormal condition:

18 $$\begin{array}{@{}rcl@{}} \sum\limits_{Q} \phi_{m}(Q) \psi_{n}(Q)=\sum \phi_{m}(Q) \hat{\phi}_{n} P_{\text{eq}}(Q)\\ = \langle \phi_{m} \hat{\phi}_{n} \rangle = \delta_{m,n} . \end{array} $$

The equilibrium time-displaced correlation function of *ϕ*_*n*_(*Q*) and $\hat {\phi }_{m}(Q)$ is given by the following:

19 $$\begin{array}{@{}rcl@{}} \langle \phi_{m}(t)\hat{\phi}_{n}(0) \rangle & =& \sum\limits_{Q} \sum\limits_{Q^{\prime}} \phi_{m}(Q) T_{t}(Q|Q^{\prime}) \hat{\phi}_{n}(Q^{\prime}) P_{\text{eq}}(Q^{\prime})\\ & =& \sum\limits_{Q} \sum\limits_{Q^{\prime}} \phi_{m}(Q) e^{-{\Gamma} t} (Q|Q^{\prime}) \hat{\phi}_{n}(Q^{\prime}) P_{\text{eq}}(Q^{\prime})\\ & =& \sum\limits_{Q} \sum\limits_{Q^{\prime}} \phi_{m}(Q) e^{-{\Gamma} t}(Q|Q^{\prime}) \psi_{n}(Q^{\prime})\\ & =& \sum\limits_{Q} \phi_{m}(Q) e^{-\lambda_{n} t}\psi_{n}(Q)\\ &= &\delta_{m,n}e^{-\lambda_{n} t}, \end{array} $$

where *T*_*t*_(*Q*|*Q*^′^) = *e*^−Γ*τ*^(*Q*|*Q*^′^) is the conditional probability that the system is found at time *t* at *Q* given that the system is at *Q*^′^ at time 0.

If two quantities *A*(*Q*) and *B*(*Q*) are expanded as

20 $$ A(Q) =\sum\limits_{n} a_{n} \phi_{n} (Q)~ \text{and}~ B(Q) =\sum\limits_{n} \hat{b}_{n} \hat{\phi}_{n} (Q),  $$

then the time correlation function of *A* and *B* in the equilibrium state is given by

21 $$ \langle A(t) B(0) \rangle =\sum\limits_{n} a_{n} \hat{b}_{n} \exp(-\lambda_{n} t).  $$

Thus, in terms of *ϕ*_*n*_(*Q*) and $\hat {\phi }_{n}(Q)$, the correlation function 〈*A*(*t*)*B*(0)〉 is decomposed into a sum of exponentially relaxing contributions. Therefore, we use two sets of functions, {*ϕ*_*n*_(*Q*)} and $\{ \hat {\phi }_{n}(Q) \}$, as relaxation modes, and refer to {*λ*_*n*_} as their relaxation rates. The relaxation modes and rates are given as left eigenfunctions and eigenvalues of the time evolution operator of the master equation of the system, respectively.

## RMA

### RMA with a single evolution time, *t*_0_

RMA approximately estimates slow relaxation modes and rates from trajectories obtained from simulations. Herein, we explain how to obtain the slow relaxation modes and rates. The point of this method is that we consider the variational problem, which is equivalent to the eigenvalue problem of the time evolution operator, and choose an appropriate trial function in order to estimate the slow relaxation modes and rates in the system.

We consider the equations for the conditional probability:

22 $$ \sum\limits_{Q} \phi_{n}(Q) T_{\tau}(Q|Q^{\prime})=e^{-\lambda_{n} \tau} \phi_{n}(Q^{\prime}),  $$

23 $$ \sum\limits_{Q^{\prime}} T_{\tau}(Q|Q^{\prime})\psi_{n}(Q^{\prime})=e^{-\lambda_{n} \tau} \psi_{n}(Q).  $$

The eigenvalue problem in Eqs. 22 and 23 is equivalent to the variational problem

24 $$ \delta \mathcal{R}= 0  $$

with

25 $$ \mathcal{R}[\phi_{n}]=\displaystyle \frac{ \langle \phi_{n}(\tau)\hat{\phi}_{n}(0) \rangle} { \langle \phi_{n}(0)\hat{\phi}_{n}(0) \rangle},  $$

and the stationary value of $\mathcal {R}$ gives the eigenvalue exp(−*λ*_*n*_*τ*). RMA treats the variational problem of Eqs. 24 and 25 using trial functions instead of the eigenvalue problem of Eqs. 22 and 23. To choose the trial function given by a linear combination of important relevant quantities, we can evaluate the relaxation modes and rates from simulation data.

Herein, we consider a biopolymer composed of *N* atoms and only treat the coordinates, because the velocities have faster relaxations (∼ picosecond order) than coordinates in protein systems. We assume that ***R*** is a 3*N*-dimensional column vector that consists of a set of atomic coordinates relative to their average coordinates

26 $$ {\boldsymbol{R}}^{\mathrm{T}}=({{\boldsymbol{r}}_{1}^{\prime}}^{\mathrm{T}},{{\boldsymbol r}_{2}^{\prime}}^{\mathrm{T}},\ldots,{{\boldsymbol r}_{N}^{\prime}}^{\mathrm{T}} ) =(x_{1}^{\prime},y_{1}^{\prime},z_{1}^{\prime},\ldots,x_{N}^{\prime},y_{N}^{\prime},z_{N}^{\prime}),  $$

with

27 $$ {\boldsymbol r}_{i}^{\prime}={\boldsymbol r}_{i} - \langle{\boldsymbol r}_{i} \rangle ,  $$

where ***r***_*i*_ is the coordinate of the *i* th atom of the biopolymer in the center-of-mass coordinate system, and 〈***r***_*i*_〉 is its average. Note that because we consider the coordinates only, $\hat {\phi }_{n}(Q)=\phi _{n}(\epsilon Q)=\phi _{n}(Q)$ holds.

In RMA, we use the following function as an approximate relaxation mode:

28 $$ X_{p}(Q)=\sum\limits_{i = 1}^{3N}f_{p,i}R_{i}(t_{0}/2;Q),  $$

with

29 $$ R_{i}(t;Q)=\sum\limits_{Q^{\prime}}R_{i}(Q^{\prime})T_{t}(Q^{\prime}|Q).  $$

Here, *R*_*i*_(*Q*) is the *i* th component of ***R***. The quantity *R*_*i*_(*t*;*Q*) is the expectation value of *R*_*i*_ after a period *t* starting from a state *Q* and satisfies *R*_*i*_(*t*;*Q*)|_*t*= 0_ = *R*_*i*_(*Q*). The parameter *t*_0_ is introduced in order to reduce the relative weight of the faster modes contained in ***R***, and it is expected that Eq. 28 becomes a better approximation as *t*_0_ becomes larger.

For the trial function (28), $\mathcal {R}$ defined by Eq. 25 is given by

30 $$ \mathcal{R}[X_{p}]= \frac{\sum\limits_{i = 1}^{3N} \sum\limits_{j = 1}^{3N} f_{p,i}C_{i,j}(t_{0}+\tau)f_{p,j}} {\sum\limits_{i = 1}^{3N} \sum\limits_{j = 1}^{3N} f_{p,i}C_{i,j}(t_{0})f_{p,j}},  $$

where *C*_*i*, *j*_(*t*) is a component of a 3*N* × 3*N* symmetric matrix *C*(*t*) defined by

31 $$ C_{i,j}(t)=\langle R_{i}(t)R_{j}(0) \rangle.  $$

Then, the variational problem of Eq. 25 becomes a generalized eigenvalue problem

32 $$ \sum\limits_{j = 1}^{3N}C_{i,j}(t_{0}+\tau)f_{p,j}=\exp(-\lambda_{p} \tau) \sum\limits_{j = 1}^{3N}C_{i,j}(t_{0})f_{p,j} .  $$

The orthonormal condition of Eq. 18 for *X*_*p*_ is written as

33 $$ \sum\limits_{i = 1}^{3N} \sum\limits_{j = 1}^{3N} f_{p,i}C_{i,j}(t_{0})f_{p,j}=\delta_{p,q}.  $$

Equations 32 and 33 determine the relaxation rates *λ*_*p*_ and the corresponding relaxation modes *f*_*p*, *i*_. We chose the indices of *λ*_*p*_ so that 0 < *λ*_1_ ≤ *λ*_2_ ≤⋯ holds. Here, the relation

34 $$ T_{t}(Q|Q^{\prime})P_{\text{eq}}(Q^{\prime})=T_{t}(Q^{\prime}|Q)P_{\text{eq}}(Q),  $$

which is equivalent to the detailed balance condition of Eq. 14 with *𝜖Q* = *Q*, and the Markovian property

35 $$ \sum\limits_{Q^{\prime}}T_{t_{1}}(Q|Q^{\prime})T_{t_{2}}(Q^{\prime}|Q^{\prime \prime})=T_{t_{1}+t_{2}}(Q|Q^{\prime \prime})  $$

are used.

The inverse transformation of Eq. 28 is given by

36 $$ R_{i}(t_{0}/2;Q)=\sum\limits_{p = 1}^{3N} g_{i,p}X_{p}(Q)  $$

with

37 $$ g_{i,p}=\sum\limits_{j = 1}^{3N}C_{i,j}(t_{0})f_{p,j}.  $$

The time correlation functions of *R*_*i*_ are reproduced by

38 $$\begin{array}{@{}rcl@{}} \langle R_{i}(t) R_{j}(0) \rangle & =& \sum\limits_{p} \sum\limits_{q} g_{i,p} g_{j,q} \left\langle X_{p} \left( t - t_{0}\right) X_{q}(0) \right\rangle,\\ & \simeq& \sum\limits_{p} g_{i,p}g_{j,p}\exp \left[-\lambda_{p} \left( t-t_{0}\right) \right],\\ & =&\sum\limits_{p} \tilde{g}_{i,p} \tilde{g}_{j,p} \exp(-\lambda_{p} t ), \end{array} $$

for *t* ≥ *t*_0_. Here,

39 $$ \tilde{g}_{i,p}=g_{i,p}\exp(\lambda_{p} t_{0}/2).  $$

Because we are considering position coordinates only, the detailed balance condition yields the following consequences: *C*(*t*) is a symmetric matrix, *C*_*i*, *j*_(*t*) = *C*_*j*, *i*_(*t*); {*λ*_*p*_} are real and positive, which corresponds to pure relaxation. We refer to this method as the “RMA method with a single evolution time,” which is *t*_0_/2.

In practice, the time correlation matrices for the two different times are calculated through simulations. Then, by solving the generalized eigenvalue problem, {*λ*_*p*_} and {*X*_*p*_} are obtained from the eigenvalues and eigenvectors, respectively. To examine the validity of the present analysis, the autocorrelation functions *C*_*i*, *i*_(*t*) are reconstructed from the estimated eigenvalues and eigenvectors and are compared with those directly calculated via simulation.

Herein, we comment on the trial function. When RMA was first introduced to a spin system, states of spins on a lattice were used as the trial function (Takano and Miyashita (1995)). When RMA was first introduced to polymer systems, the coordinates of polymers were used as the trial function (Koseki et al. (1997); Hirao et al. (1997)). In polymer systems, the Rouse modes, which were derived from the theory of polymer physics (Doi and Edwards 1986), correspond to the relaxation modes. Rouse modes are given as linear combinations of coordinates. Thus, when RMA was applied to polymer systems, the modes obtained by RMA were compared with the Rouse modes. In protein systems, PCA using coordinates has been widely used. In PCA, the eigenvalue problem of the covariance matrix of coordinates is solved. Therefore, when we first applied RMA to a hetero polymer system (protein system), it seemed to be better to use coordinates as trial functions. The results of RMA and PCA were directly compared with each other. Recently, we have proposed to use physical quantities with slow motions as the trial functions and PCRMA and two-step RMA have been introduced (see the “Improvement of RMA” section). However, RMA using coordinates as the trial functions has an advantage that we can easily convert the information on the slow relaxation modes to the information in coordinate space.

### RMA for protein systems

In homopolymer systems, relaxation of the positions of a polymer relative to the center of the mass is investigated. This means that the translational degrees of freedom are removed from the coordinates of the polymer. Because the rotational degrees of freedom remain, the rotational relaxation of the polymer is observed as slow relaxations. In protein systems, it is of interest to evaluate fluctuations of the conformations of a biomolecule around its average conformation. Thus, the translational and rotational degrees of freedom are removed from the sampled conformations of a biomolecule. In practice, treatment of the generalized-eigenvalue problem for removing the translational degrees of freedom in the homopolymer system was given by Koseki et al. (1997). Herein, we explain how to treat the generalized eigenvalue problem for removing the translational and rotational degrees of freedom when using the coordinates for the trial function (Mitsutake et al. 2011). The point of this process is that the generalized eigenvalue problem for real symmetric matrices can be easily solved numerically if the matrices are positive definite. Therefore, we shift the zero eigenvalues to finite positive values without changing the other eigenvalues and the corresponding eigenvectors.

**Fig. 1:**
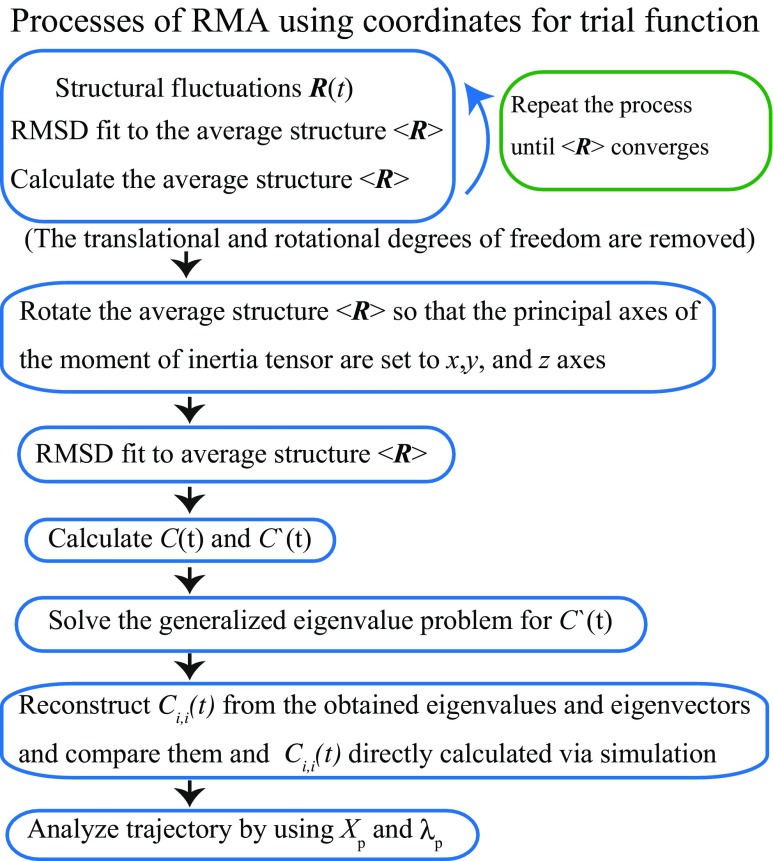
Schematic illustration of the RMA process using the coordinate ***R*** for the trial function

A schematic illustration of the process for RMA using coordinates for the trial function is shown in Fig. 1. First, we remove the translational and rotational degrees of freedom as well as conduct PCA (Eckart 1935; McLachlan 1979). After the average structure converges, the origin of the coordinate system is chosen to be the center of the mass of the average positions, 〈***r***_*i*_〉 with *i* = 1,…,*N*, and the axes of the coordinate system are chosen to be the principal axes of the moment of the inertia tensor of the average positions. We calculate $C_{i,j}(t)= \frac {C_{i,j}(t)+C_{j,i}(t)}{2}$ and *C*^′^(*t*):

40 $$\begin{array}{@{}rcl@{}} C^{\prime}(t)&=& C(t)+\sum\limits_{\alpha=x,y,z} \exp(-\lambda_{\alpha}^{\text{tr}}(t-t_{0})) {\boldsymbol d}_{\alpha}^{\text{tr}} {{\boldsymbol d}_{\alpha}^{\text{tr}}}^{\mathrm{T}}\\ &&+ \sum\limits_{\alpha=x,y,z} \exp(-\lambda_{\alpha}^{\text{rot}}(t-t_{0})) {\boldsymbol d}_{\alpha}^{\text{rot}} {{\boldsymbol d}_{\alpha}^{\text{rot}}}^{\mathrm{T}}, \end{array} $$

where ${\boldsymbol d}_{x}^{\text {tr}}$, ${\boldsymbol d}_{y}^{\text {tr}}$, and ${\boldsymbol d}_{z}^{\text {tr}}$ are unit vectors given by

41 $$\begin{array}{@{}rcl@{}} {\boldsymbol d}_{x}^{\text{tr}}&=& \frac{1}{\sqrt{N}} (1,0,0,1,0,0,\cdots,1,0,0)^{\mathrm{T}},\\ {\boldsymbol d}_{y}^{\text{tr}}&=& \frac{1}{\sqrt{N}} (0,1,0,0,1,0,\cdots,0,1,0)^{\mathrm{T}},\\ {\boldsymbol d}_{z}^{\text{tr}}&=& \frac{1}{\sqrt{N}} (0,0,1,0,0,1,\cdots,0,0,1)^{\mathrm{T}}, \end{array} $$

and ${\boldsymbol d}_{x}^{\text {rot}}$, ${\boldsymbol d}_{y}^{\text {rot}}$, and ${\boldsymbol d}_{z}^{\text {rot}}$ are unit vectors given by

42 $$\begin{array}{@{}rcl@{}} {\boldsymbol d}_{x}^{\text{rot}}&=& \frac{1}{ \sqrt{ \sum\limits_{i = 1}^{N} (\langle z_{i} \rangle^{2} + \langle y_{i} \rangle^{2})} }\\ &&\times (0,-\langle z_{1} \rangle ,\langle y_{1} \rangle, 0,-\langle z_{2} \rangle ,\langle y_{2} \rangle ,\cdots, 0,-\langle z_{N} \rangle ,\langle y_{N} \rangle )^{\mathrm{T}},\\ {\boldsymbol d}_{y}^{\text{rot}}&=& \frac{1}{ \sqrt{\sum\limits_{i = 1}^{N} (\langle z_{i} \rangle^{2} + \langle x_{i} \rangle^{2})}}\\ & &\times (\langle z_{1} \rangle ,0,-\langle x_{1} \rangle, \langle z_{2} \rangle ,0,-\langle x_{2} \rangle ,\cdots, \langle z_{N} \rangle ,0,-\langle x_{N} \rangle )^{\mathrm{T}}, \text{and}\\ {\boldsymbol d}_{z}^{\text{rot}}&=& \frac{1}{ \sqrt{\sum\limits_{i = 1}^{N} (\langle y_{i} \rangle^{2} + \langle x_{i} \rangle^{2})}}\\ &&\times (-\langle y_{1} \rangle ,\langle x_{1} \rangle ,0, -\langle y_{2} \rangle ,\langle x_{2} \rangle ,0,\cdots, -\langle y_{N} \rangle ,\langle x_{N} \rangle ,0)^{\mathrm{T}}. \end{array} $$

The values of $\lambda _{\alpha }^{\text {tr}}$ and $\lambda _{\alpha }^{\text {rot}}$ are usually set to zero. These unit vectors satisfy the following relations:

43 $$ {\boldsymbol d}_{\alpha}^{a} \cdot {\boldsymbol d}_{\beta}^{b} ={{\boldsymbol d}_{\alpha}^{a}}^{\mathrm{T}} \: {\boldsymbol d}_{\beta}^{b} =\delta_{\alpha,\beta} \delta_{a,b} $$

and

44 $$ C(t){\boldsymbol d}_{\alpha}^{a}= 0, $$

where *α*, *β* = *x*, *y*, *z* and *a*, *b* = tr,rot. Then, we solve the generalized eigenvalue problem for *C*^′^(*t*_0_ + *τ*) and *C*^′^(*t*_0_), $ C^{\prime }(t_{0}+\tau ) {\boldsymbol v}^{\prime }_{p} = \exp (-\lambda ^{\prime }_{p} \tau ) C^{\prime }(t_{0}) {\boldsymbol v}^{\prime }_{p} $, with the orthonormal condition $ {{\boldsymbol v}^{\prime }_{p}}^{\mathrm {T}} C^{\prime }(t_{0}) {\boldsymbol v}^{\prime }_{q} = \delta _{p,q} $. The unit vectors ${\boldsymbol d}^{a}_{\alpha }$ are eigenvectors of this generalized eigenvalue problem with eigenvalues $\exp (-\lambda _{\alpha }^{a}\tau )$. We denote ${\boldsymbol f}^{\prime }_{p}$ as the eigenvectors other than ${\boldsymbol d}_{\alpha }^{a}$. Because $ {{\boldsymbol d}_{\alpha }^{a}}^{\mathrm {T}}C^{\prime }(t){\boldsymbol f}^{\prime }_{p} = \exp (-\lambda ^{a}_{\alpha }(t-t_{0})) {{\boldsymbol d}_{\alpha }^{a}}^{\mathrm {T}}{\boldsymbol f}^{\prime }_{p} = 0 $ , $ C^{\prime }(t){\boldsymbol f}^{\prime }_{p} = C(t) {\boldsymbol f}^{\prime }_{p} $ holds. Therefore, ${\boldsymbol f}^{\prime }_{p}$ are identical with the eigenvectors ***f***_*p*_ = (*f*_*p*,1_,*f*_*p*,2_,…,*f*_*p*,3*N*_)^T^ of the generalized-eigenvalue problem for *C*(*t*_0_ + *τ*) and *C*(*t*_0_) with the same eigenvalues exp(−*λ*_*p*_*τ*). Thus, ***f***_*p*_ and exp(−*λ*_*p*_*τ*) can be obtained by solving the generalized eigenvalue problem for *C*^′^(*t*_0_ + *τ*) and *C*^′^(*t*_0_), which are real symmetric positive definite matrices.

After obtaining relaxation modes and rates, we confirm whether or not the slow relaxation modes and rates obtained using *τ* and *t*_0_ are appropriate. For this purpose, the convergences of slow relaxation times as a function of *τ* are examined. The autocorrelation functions *C*_*i*, *i*_(*t*) are reconstructed from the estimated eigenvalues and eigenvectors and are compared with those directly calculated via simulation (especially the slow relaxation behavior). After examining the validity, we use the obtained relaxation modes and rates for analysis.

## Improvement of RMA

### Selection of *τ* and *t*_0_ and relevant quantities for the trial function

**Fig. 2:**
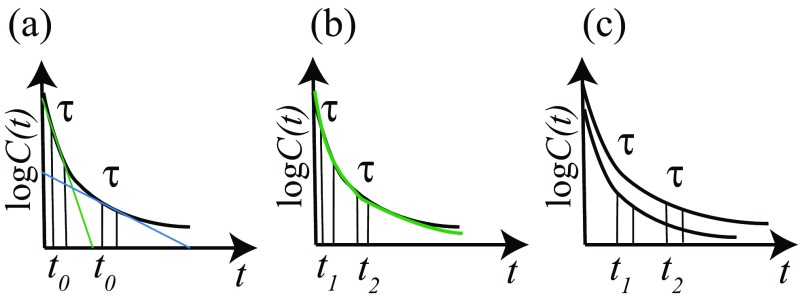
Schematic illustration of RMA with a single evolution time *t*_0_ (**a**), and multiple evolution times (1) using *t*_1_ and *t*_2_ (**b**) and (2) using *t*_*i*_ (**c**)

The relaxation times {1/*λ*_*p*_} and the {*X*_*p*_} obtained via RMA depend on the manner in which *t*_0_ and *τ* are selected in practice. For simplification, we here consider the case of one physical quantity, *R*. From the variational problem of Eqs. 24 and 25, the relaxation time 1/*λ* is obtained from the gradient of the straight line connecting two points at *t* = *t*_0_ and *t* = *t*_0_ + *τ* in the semi-log plot of the correlation function *C*(*t*) = 〈*R*(*t*)*R*(0)〉−〈*R*〉^2^ versus *t*, as shown in Fig. 2a. If the time correlation function of the physical quantity contains several {1/*λ*_*p*_}, and if we choose *t*_0_ = 0 (tICA case) or a small *t*_0_ and small *τ*, as shown in Fig. 2a (green line), the obtained 1/*λ* does not correspond to the slow relaxation behavior of log *C*(*t*) at long times. To investigate the slow relaxation, we wish to choose values of *t*_0_ and *τ* that are as large as possible, as shown in Fig. 2a (blue line). However, the choice of a longer *t*_0_ and *τ* is also limited, because of the decreasing accuracy of the time correlation function over long time periods. Therefore, we must choose the appropriate *t*_0_ and *τ*.

We can improve the RMA explained above by using two different approaches: introduction of multiple evolution times and using the different relevant physical quantities obtained from coordinates (and velocities) for the trial function. For the first improvement, we describe two types of methods with multiple evolution times, as shown in Fig. 2b, c. (The detailed descriptions are given by Nagai et al. (2013), Natori and Takano (2017), and Karasawa et al. (2017).) For the second improvement, we describe the PCRMA (Nagai et al. 2013), in which the relevant physical quantities for the trial function are given by the PC modes with large structural fluctuations and the two-step RMA (Natori and Takano 2017; Karasawa et al. 2017), which are in turn given by the slowest relaxation modes roughly obtained by RMA. Moreover, the MSRMA (Mitsutake and Takano 2015) is also proposed. We will describe these two improved RMAs in detail below.

### RMA with multiple evolution times

#### RMA with multiple evolution times *t*_1_ and *t*_2_(1)

In this method, the following trial functions are used as approximate relaxation modes:

45 $$ X_{p}(Q)=\sum\limits_{i = 1}^{3N}f^{1}_{p,i}R_{i}(t_{1}/2;Q) +\sum\limits_{i = 1}^{3N}f^{2}_{p,i}R_{i}(t_{2}/2;Q).  $$

Note that two evolution times, *t*_1_/2 and *t*_2_/2, are used instead of a single evolution time, *t*_0_/2. Because the contributions of faster modes in ***R*** time-evolved for *t*_1_/2 and those for *t*_2_/2 are different, the approximate relaxation modes can extract the faster modes, which cannot be extracted by the approximate relaxation modes using a single evolution time (see Fig. 2b). Using Eq. 45 as a trial function for the variational problem, the following generalized eigenvalue problem is obtained:

46 $$ \sum\limits_{j = 1}^{6N}C_{i,j}(t_{0}+\tau)f_{p,j}=\exp(-\lambda_{p} \tau) \sum\limits_{j = 1}^{6N}C_{i,j}(t_{0})f_{p,j} ,  $$

with ${\boldsymbol f}_{p} = ({{\boldsymbol f}^{1}_{p}}^{\mathrm {T}},{{\boldsymbol f}^{2}_{p}}^{\mathrm {T}})^{\mathrm {T}}$. Here, *C*(*t*) is a 6*N* × 6*N* matrix defined by

47 $$ C(t)= \left( \begin{array}{ll} C^{1,1}(t) & C^{1,2}(t) \\ C^{2,1}(t) & C^{2,2}(t) \end{array} \right),  $$

and $C^{\mu _{1},\mu _{2}}(t) $ is an 3*N* × 3*N* matrix defined by

48 $$ C^{\mu_{1},\mu_{2}}_{i,j}(t)= \left< R_{i}\left( \frac{t_{\mu_{1}}}{2}+ \frac{t_{\mu_{2}}}{2}+t\right) R_{j}(0) \right>,  $$

where *μ*_1_, *μ*_2_ = 1 or 2. The orthonormal condition is written as

49 $$ \sum\limits_{i = 1}^{6N} \sum\limits_{j = 1}^{6N} f_{p,i}C_{i,j}(0)f_{p,j}=\delta_{p,q}.  $$

The inverse transformation of Eq. 45 is given by

50 $$\begin{array}{@{}rcl@{}} R_{i}(t_{1}/2;Q)= \sum\limits_{p = 1}^{6N} g^{1}_{i,p}X_{p}(Q)\\ R_{i}(t_{2}/2;Q)=\displaystyle \sum\limits_{p = 1}^{6N} g^{2}_{i,p}X_{p}(Q) \end{array} $$

with

51 $$ g_{i,p}=\sum\limits_{j = 1}^{6N}C_{i,j}(0)f_{p,j},  $$

where ${\boldsymbol g}_{p} = \left ({{\boldsymbol g}^{1}_{p}}^{\mathrm {T}},{{\boldsymbol g}^{2}_{p}}^{\mathrm {T}}\right )^{\mathrm {T}}$. The time correlation functions of *R*_*i*_ are reproduced by

52 $$ \langle R_{i}(t) R_{j}(0) \rangle \simeq \sum\limits_{p = 1}^{6N} \tilde{g}^{av}_{i,p}\tilde{g}^{av}_{j,p}\exp \left( -\lambda_{p} t \right),  $$

where

53 $$ \tilde{g}^{av}_{i,p}=(\exp(\lambda_{p} t_{1}/2)g^{1}_{i,p}+\exp(\lambda_{p} t_{2}/2)g^{2}_{i,p})/2.  $$

#### RMA with multiple evolution times *t*_*i*_ (2)

When the relevant physical quantities ***R*** in the trial function exhibit different relaxations, it is preferable to use different evolution times for the different physical quantities, as shown in Fig. 1c. That is, if we know the characteristic time scales of the relevant physical quantities, we can choose a specific evolution time *t*_*i*_ for each relevant physical quantity *R*_*i*_ based on its characteristic time scale. This RMA method is referred to as “RMA with multiple evolution times {*t*_*i*_/2}.” In this method, we use the following trial function:

54 $$ X_{p}(Q)=\sum\limits_{i = 1}^{3N}f_{p,i}R_{i}(t_{i}/2;Q).  $$

The parameter *t*_*i*_ is introduced in order to reduce the relative weight of the faster modes contained in *R*_*i*_. Further, it is expected that Eq. 54 would yield a superior approximation for larger *t*_*i*_ values.

The variational problem becomes a generalized-eigenvalue problem:

55 $$ \sum\limits_{j = 1}^{3N}C_{i,j} \left( \frac{t_{i}+t_{j}}{2}+\tau\right) f_{p,j}=\exp(-\lambda_{p} \tau) \sum\limits_{j = 1}^{3N}C_{i,j} \left( \frac{t_{i}+t_{j}}{2} \right) f_{p,j} .  $$

Here, *C*_*i*, *j*_(*t*) = 〈*R*_*i*_(*t*)*R*_*j*_(0)〉 and the orthonormal condition for *X*_*p*_ is expressed as

56 $$ \sum\limits_{i = 1}^{3N} \sum\limits_{j = 1}^{3N} f_{p,i} C_{i,j} \left( \frac{t_{i}+t_{j}}{2} \right) f_{q,j}=\delta_{p,q}.  $$

Equations 54, 55, and 56 determine the relaxation rates *λ*_*p*_ and the corresponding relaxation modes. We chose the indices of *λ*_*p*_ such that 0 < *λ*_1_ ≤ *λ*_2_ ≤⋯ holds. The inverse transformation of Eq. 54 is given by

57 $$ R_{i}(t_{i}/2;Q)=\sum\limits_{p = 1}^{3N-6} g_{i,p}X_{p}(Q),  $$

with

58 $$ g_{i,p}=\sum\limits_{j = 1}^{3N} C_{i,j}\left( \frac{t_{i}+t_{j}}{2}\right) f_{p,j}.  $$

The time correlation functions of *R*_*i*_ are given by

59 $$\begin{array}{@{}rcl@{}} \langle R_{i}(t) R_{j}(0) \rangle &\!\,=\,\!& \sum\limits_{p} \!\sum\limits_{q} g_{i,p} g_{j,q} \left\langle X_{p} \left( t - \frac{t_{i}+t_{j}}{2}\right) X_{q}(0) \right\rangle,\\ &\!\!\!\simeq\!\!\!& \sum\limits_{p} g_{i,p}g_{j,p}\exp \left[-\lambda_{p} \left( t-\frac{t_{i}+t_{j}}{2}\right) \right],\\ &\!\,=\,\!&\sum\limits_{p} \tilde{g}_{i,p} \tilde{g}_{j,p} \exp(-\lambda_{p} t ), \end{array} $$

for *t* ≥ (*t*_*i*_ + *t*_*j*_)/2. Here,

60 $$ \tilde{g}_{i,p}=g_{i,p}\exp(\lambda_{p} t_{i}/2).  $$

### RMAs to automatically reduce the degrees of freedom of relevant quantities for the trial function

RMA requires relatively high statistical precision of the time correlation matrices because of treatment for the generalized eigenvalue problem; thus, it is difficult for RMA to handle a large number of degrees of freedom directly. We must therefore reduce the number of degrees of freedom automatically.

**Fig. 3:**
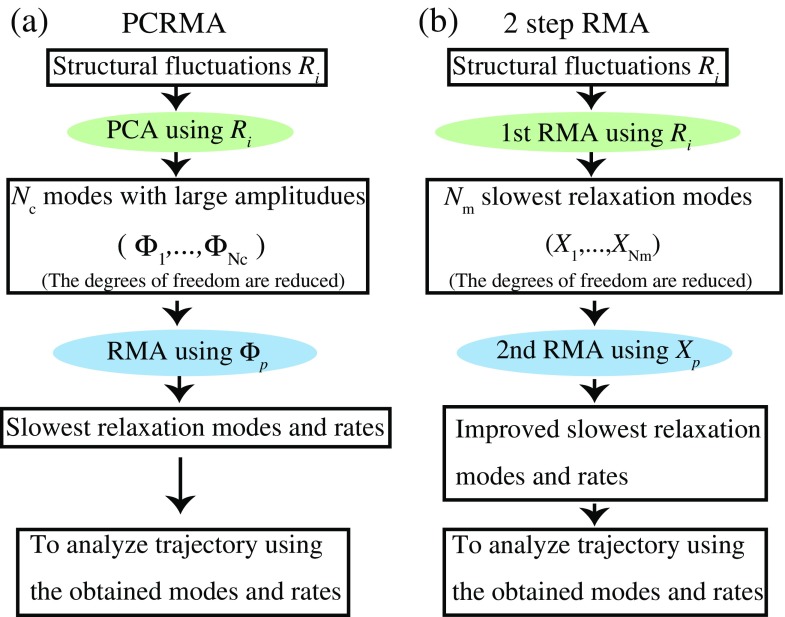
Schematic illustration of PCRMA (**a**) and two-step RMA (**b**)

In an original RMA, the coordinates (and velocity) are used for the trial function. The results may change depending on which relevant quantities are used for the trial function because their correlation functions are fitted using *t*_0_ and *τ*. (For the Markov state model, the dependence of relaxation times on the selection of states is discussed in Swope et al. (2004) and Pérez-Hernández et al. (2013).) It is better to use the relevant quantities that include the slow behavior. For the second improvement, we describe the PCRMA in which the relevant quantities are given by the PC modes with large structural fluctuations, and the two-step RMA in which the quantities are given by the slowest relaxation modes roughly obtained by the first RMA. A schematic illustration of PCRMA and two-step RMA is given in Fig. 3.

#### PCRMA

To apply RMA to a protein system by reducing its degrees of freedom, we proposed an improved method, which is referred to as the PCRMA method (Nagai et al. 2013). In this method, PCA is carried out first, and then, RMA is applied to a small number of principal components with large fluctuations (**Φ** =(${\Phi }_{1},{\Phi }_{2},\cdots ,{\Phi }_{N_{c}})^{T}$). We use the following function as an approximate relaxation mode:

61 $$ X_{p}(Q)=\sum\limits_{i = 1}^{N_{c}}f_{p,i}{\Phi}_{i}(t_{0}/2;Q).  $$

**Fig. 4:**
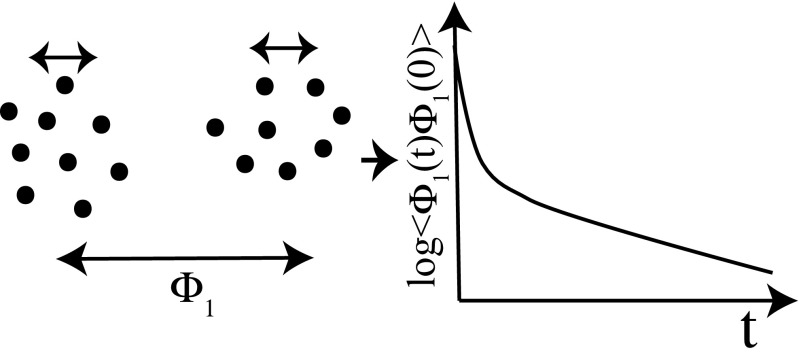
Schematic illustrationfor PCRMA

Because the degrees of freedom is reduced to *N*_*c*_ and the relevant quantities with large variance tend to have slow relaxations, the slow relaxation times can be estimated by setting *t*_0_ and *τ* as large values. Note that because the selected principal components also contain faster relaxation modes, as shown in Fig. 4, Nagai et al. (2013) also combined PCRMA with the RMA using multiple evolution times (1) explained above. Note that in PCRMA, if the *N*_*c*_th or more PC modes (with relatively small fluctuations) have slow relaxation, the slow behaviors may not be extracted; thus, there is a possibility that the slow relaxations would not be estimated with small structural fluctuations.

#### Two-step RMA

Using a similar process to that of PCRMA, we proposed a two-step RMA method (Natori and Takano 2017; Karasawa et al. 2017). Based on our experience, the slow {*X*_*p*_} obtained from the conventional RMA with small *t*_0_ and *τ* contains the true slow {*X*_*p*_} (Mitsutake et al. 2011), although the {1/*λ*_*p*_} values are underestimated. The slow relaxation modes obtained by the first RMA may contain the true slow relaxation modes. Thus, we use the slow relaxation modes roughly obtained from the first RMA as the relevant quantities for the trial function. In this technique, RMA with a single evolution time using small *t*_0_ and *τ* is implemented first, and {*X*_*p*_} and {*λ*_*p*_} are roughly estimated. We then apply the second RMA to a small number of the obtained slowest {*X*_*p*_}. We denote the number of {*X*_*p*_} used in the second RMA as *N*_m_. In the second RMA, we also use the previously presented technique of RMA with multiple evolution times (2), because the characteristic time scales of the {*X*_*p*_} obtained from the first RMA are roughly given by the relaxation times {1/*λ*_*p*_}. In the second RMA, we use the following trial function:

62 $$ X_{u}^{\prime}(Q) = \sum\limits_{p = 1}^{N_{\mathrm{m}}} f_{u,p}^{\prime}X_{p}(t_{p}^{\prime}/2;Q).  $$

Here, *X*_*p*_(*Q*) is the relaxation mode obtained from the first RMA and $t_{p}^{\prime }$ is determined from 1/*λ*_*p*_. A detailed explanation is given by Natori and Takano (2017) and Karasawa et al. (2017).

In the second RMA, the time interval *τ* can be chosen to be large, because the number of degrees of freedom is reduced and the physical quantities {*X*_*p*_} exhibit slow relaxations. Using the second RMA, the estimation accuracy of the relaxation modes and times can be improved.

### Markov state RMA

As mentioned above, in RMA, the relaxation modes and rates are given as left eigenfunctions and eigenvalues of the time evolution operator of the master equation of the system, respectively. From this point of view, RMA is related to Markov state models. Herein, we consider the relation between RMA and Markov state models and propose the new method of MSRMA.

In the simplest Markov state model, the phase space of the system, where only the position coordinates are considered, is divided into clusters (subsets) *S*_*i*_, *i* = 1,…,*n*. First, the joint probability $\bar {P}_{i,j}(\tau )=P(Q \in S_{i}, \tau ; Q \in S_{j} , 0)$ that the state of the system *Q* is in the *j* th cluster at time 0 and is in the *i* th cluster at time *τ* > 0 is calculated in a simulation. Second, the transition probability $\bar {T}_{i,j}(\tau )$ that the state of the system is found in the *i* th cluster after time *τ* starting from a state in the *j* th cluster is calculated by

63 $$ \bar{T}_{i,j}(\tau) = \bar{P}_{i,j}(\tau)/\bar{p}_{j},  $$

where $\bar {p}_{j} = P(Q \in S_{j})$ is the probability that the state of the system is found in the *j* th cluster, which is estimated in the simulation. Then, by solving the eigenvalue problem

64 $$ {\bar{\boldsymbol f}_{p}}{}^{\mathrm{T}} \bar{T}(\tau) = {\bar{\boldsymbol f}_{p}}{}^{\mathrm{T}} \bar{{\Lambda}}_{p}  $$

for the transition matrix $\bar {T}(\tau ) = (\bar {T}_{i,j}(\tau ))$, the *p* th eigenvector $\bar {\boldsymbol f}_{p}$ and its eigenvalue $\bar {{\Lambda }}_{p}$ are obtained. The eigenvector $\bar {\boldsymbol f}_{1} \propto (1,1,\ldots ,1)^{\mathrm {T}}$ corresponds to the equilibrium state and its eigenvalue $\bar {{\Lambda }}_{1} = 1$. Other eigenvectors $\bar {\boldsymbol f}_{p}$ represent structural transitions and the corresponding eigenvalues $\bar {{\Lambda }}_{p}$ give their relaxation time scales $\bar {\tau }_{p}$ as

65 $$ \bar{\tau}_{p} = - \frac{\tau}{\ln \bar{{\Lambda}}_{p}}.  $$

Note that in the Markov description, it is important that the states are defined in a kinetically meaningful way (Swope et al. 2004; Pérez-Hernández et al. 2013). We need to define the states that are classified by order parameters representing the dynamics and kinetics of the system. Even with a good choice of states, in order for a Markov description of the process to be accurate, the time interval *τ* should also be chosen carefully. In other words, for the Markov description to work, the time interval of the transition matrix *τ* must be chosen appropriately so that it is as large as the slowest relaxation time of the states. When plotting ${\bar \tau }_{p}$ as a function of *τ*, ${\bar \tau }_{p}$ slowly converges to the appropriate time scale when *τ* is increased. In addition, when a much longer *τ* than the slowest relaxation time of the states is used, the Markov state model is not expected to be accurate. Thus, we usually set the time interval *τ* to the value when the variation of *τ*_*p*_ is sufficiently flat (Swope et al. 2004; Pérez-Hernández et al. 2013).

The abovementioned procedure of the Markov state model is related to the following procedure of RMA. We consider an approximate relaxation mode given by

66 $$ \bar{X}_{p}=\sum\limits_{i = 1}^{n}f_{p,i}\delta_{i}(t_{0}/2;Q),  $$

where *δ*_*i*_(*t*;*Q*) is defined in the same way as *R*_*i*_(*t*;*Q*) in Eq. 29 from *δ*_*i*_(*Q*) given as a function of the state *Q* of the system by

67 $$ \delta_{i}(Q) = \left\{ \begin{array}{lcl} 1 & \text{for} & Q \in S_{i},\\ 0 & \text{for} & Q \notin S_{i}. \end{array} \right. $$

Then, the generalized eigenvalue problem is given by

68 $$ \sum\limits_{j} \bar{C}_{i,j}(t_{0}+\tau)\bar{f}_{p,j} = \mathrm{e}^{-\bar{\lambda}_{p} \tau} \sum\limits_{j} \bar{C}_{i,j}(t_{0})\bar{f}_{p,j},  $$

with

69 $$ \sum\limits_{i,j} \bar{f}_{p,i} \bar{C}_{i,j}(t_{0}) \bar{f}_{q,j} =\delta_{p,q},  $$

where

70 $$ \bar{C}_{i,j}(t)= \langle \delta_{i}(t) \delta_{j}(0) \rangle.  $$

According to the definition of *δ*_*i*_(*Q*), it follows that $\bar {C}_{i,j}(t)$ is the joint probability $\bar {P}_{i,j}(t)$.

If we set *t*_0_ = 0, the generalized eigenvalue problem (68) becomes the eigenvalue problem (64) with $\bar {{\Lambda }}_{p} = \mathrm {e}^{-\bar {\lambda }_{p} \tau }$ or $\bar {\tau }_{p} = 1/\bar {\lambda }_{p}$, because $\bar {C}(0)=\text {diag}(\bar {p}_{1},\ldots ,\bar {p}_{n})$ and $\bar {C}(\tau )\bar {C}(0)^{-1} = \bar {T}(\tau )$. Thus, the Markov state model is a special case of MSRMA with *t*_0_ = 0.

Because *δ*_*i*_(*t*_0_/2;*Q*) in Eq. 66 reduces the contributions of faster modes in *δ*_*i*_(*Q*), the solutions of the generalized eigenvalue problem (68) provides better approximations to the slow relaxation modes and rates as *t*_0_ becomes larger. Therefore, the relaxation times $\bar {\tau }_{p}$ obtained by the Markov state model are expected to be improved by solving Eq. 68 with *t*_0_ > 0 rather than Eq. 64.

## Application of RMA to a system with large conformational changes

In this section, we apply RMA to a protein system simulation to show the effectiveness of RMA. The selection of order parameters in simulations is important to analyze the trajectory. PCA, which is a static analysis method, extracts large structural fluctuations from simulations, and the obtained PC mode is used to obtain the order parameters. Moreover, it has now become possible to perform long simulations such as those of unfolded and folded protein structures, and when the simulation involves large structural changes, the difference between local minimum-energy states is relatively small compared with that between the folded and unfolded states. In this case, it is difficult for PCA to extract the effective modes or order parameters to accurately identify the local minimum-energy states. By contrast, RMA extracts slow relaxation modes. It is thought that the local minimum-energy states are usually stable so that the system remains in this state for a long time during a simulation. The order parameters with slow relaxation may correspond to the directions between local minimum-energy states. Thus, slow relaxation modes may be suitable order parameters to identify local minimum-energy states and the transitions between them. To validate this concept, we applied RMA to the 10-residue peptide, chignolin in water near its folding transition temperature.

**Fig. 5:**
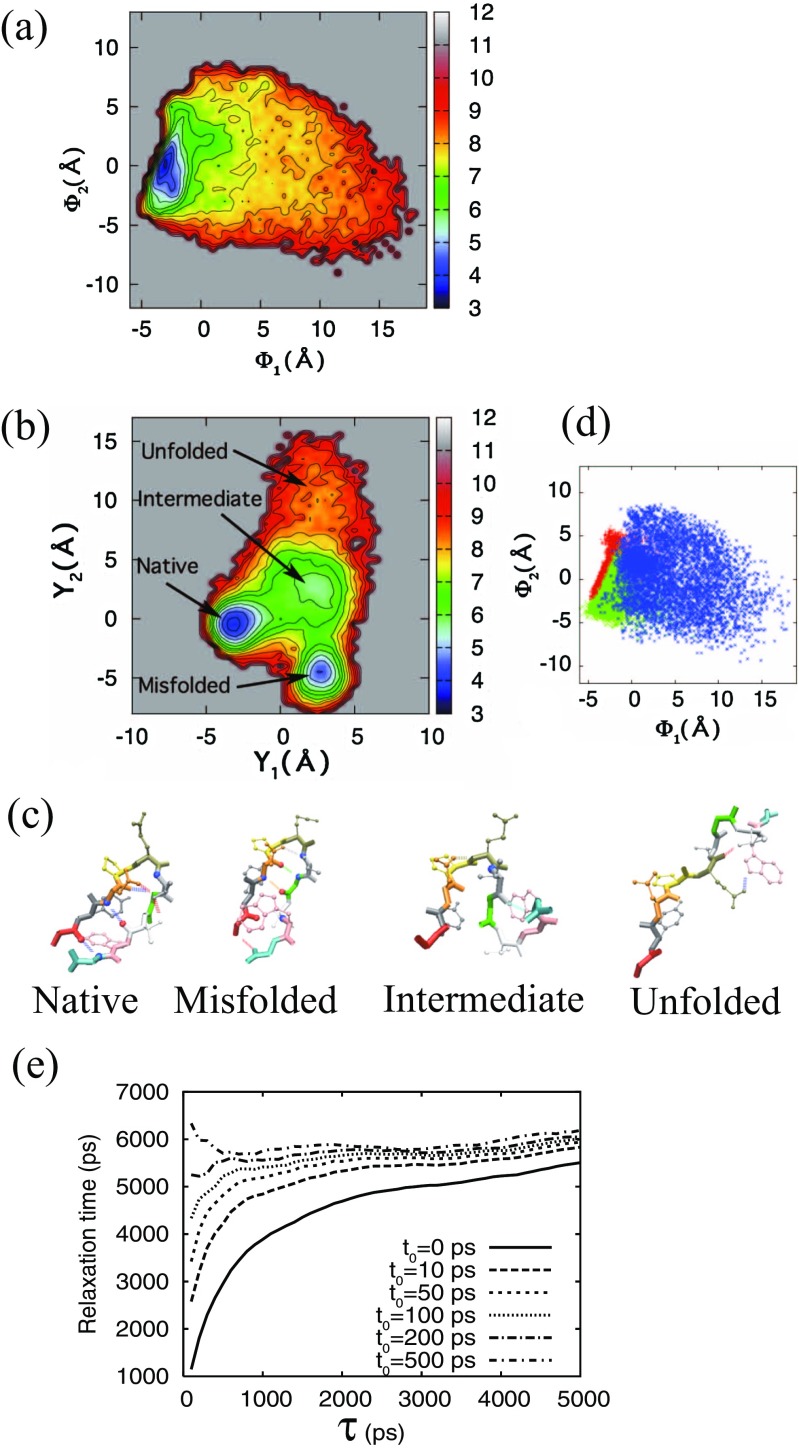
The free-energy surfaces for **a** the first PC mode Φ_1_ and the second PC mode Φ_2_, and for **b** the first slowest RM and the second slowest RM in the case of *t*_0_ = 10.0 ps and *τ* = 20.0 ps. **c** Snapshots of the native, misfolded, intermediate, and unfolded states classified by RMA, and **d** distributions for the native (red), misfolded (green), and intermediate (blue) states on the free-energy surface of the first PC mode and the second PC mode. **e** Relaxation times of the second relaxation mode obtained by MSRMA as a function of the time interval *τ*. In **e**, the line of *t*_0_ ps corresponds to the results of a simple Markov state model. The figure was reproduced from Mitsutake and Takano (2015)

The detailed results are described in Mitsutake et al. (2011). Chignolin consists of a 10-amino acid sequence, GYDPETGTWG and adopts a *β*-hairpin turn structure (Honda et al. 2004). Several simulations of chignolin have been reported to date (Satoh et al. 2006; Suenaga et al. 2007; Harada and Kitao 2011; Kührova et al. 2012; Okumura 2012). Previous research has shown that chignolin has a stable (native) and a misfolded state, which are both found as hairpin-like structures (see Fig. 5c). These two states have a common turn structure from Asp3 to Glu5 but slightly different hydrogen bond patterns. RMA requires a relatively high level of statistical precision for the time correlation matrices and therefore requires a long simulation where many transitions between local minimum-energy states occur. In addition, we sought to analyze the system with large conformational changes. Thus, we performed a 750-ns molecular dynamics simulation of chignolin in aqueous solution near the transition temperature from an extended structure (Case et al. 2014). We observed many transitions among structures, including the native, misfolded, and unfolded states, by performing the simulation at 450 K. We used the coordinates of C_*α*_ atoms on the backbone as coordinates so that the degrees of freedom were 30. After removing the translational and rotational motions from the coordinates of C_*α*_ atoms, PCA and RMA were carried out on the coordinates of C_*α*_ atoms (see Fig. 1). For RMA, we set *t*_0_ and *τ* to 10.0 and 20.0 ps, respectively.

Figure 5 shows the free-energy surfaces obtained from PCA (a) and RMA (b). From the free-energy surface of PCA, the native and misfolded states were not distinguished because the conformational difference between them is much smaller than the conformational fluctuations of the system (the third PC mode distinguished the native and misfolded states). By contrast, in RMA, the transition between the native and misfolded structures is slow, and the slowest relaxation mode was found to be the axis distinguishing them. This analysis showed that the slow relaxation mode is a good order parameter to distinguish the native and misfolded structure. Interestingly, we could also identify the intermediate structure. By extracting the structures in the center part of the free-energy surface shown in Fig. 5b, the cluster was formed with a turn structure common to the native and misfolded structures. Because the structures at both terminals fluctuate, a cluster of intermediate structures forming a turn is also obtained, while ignoring the fast relaxing movement of both terminals. The upper part of the free-energy surface shown in Fig. 5b corresponded to the extended structure. Figure 5c shows the characteristic structures for the four states. When plotting the points for the obtained intermediate structure on the free-energy surface of PCA in Fig 5d, the points were distributed widely because both terminals fluctuate. Thus, RMA can identify the characteristic structure, even when it is only partially formed. From the free-energy surface obtained by RMA, it is clarified that chignolin folds to the native or misfolded structures through the intermediate (turn) structure from the extended structures.

Because the structures were classified into a smaller number of states using the free-energy surface obtained by RMA, we then applied the Markov state model and MSRMA to analyze these four states: native, misfolded, intermediate, and unfolded states. Figure 5e shows the relaxation time *τ*_*p*_ = 1/*λ*_*p*_ obtained by MSRMA as a function of *τ* when *t*_0_ = 0,10,50,100,200, and 500 ps. Because the first eigenvector corresponds to the steady state with infinite relaxation time *τ*_1_ = *∞*, we show the second slowest relaxation times. The line of *t*_0_ = 0 corresponds to the results of a simple Markov state model. In the case of *t*_0_ = 0, the *τ*_*p*_ values slowly approach the appropriate time scale, i.e., the values for plateau regions or peak values of the solid lines, when *τ* is increased. For the lines of *t*_0_ > 0, the values of *τ*_*p*_ quickly approach the appropriate time scale, i.e., those corresponding to the values for plateau regions or peak values. Thus, the slow relaxation times can be improved when applying MSRMA with *t*_0_ > 0, which is introduced to reduce the relative weight of the faster modes.

Overall, RMA can be used to effectively analyze long simulations at room temperature and is also useful for investigating systems with large conformational changes, such as intrinsically disordered proteins and protein folding.

## Conclusions

In this paper, we have reviewed the method and application of RMA, a dynamic analysis method for protein simulations. We described the definition of relaxation modes and rates, which correspond to the left eigenfunctions and eigenvalues of the time evolution operator of the master equation of the system, respectively. After providing the definition, we explained how to estimate the slow relaxation modes and rates from simulation data. We also summarized several new RMAs proposed, including RMA with multiple evolution times, PCRMA, two-step RMA, and MSRMA. Finally, to demonstrate the effectiveness of RMA, we briefly presented the analysis results of the unfolding/folding simulation of the 10-residue peptide chignolin detected near the transition temperature. The simulation results showed that the relaxation mode is a good order parameter for not only extracting the transition between the native state and misfolded state but also for identifying the intermediate state, which is partially folded. This suggests that RMA is suitable to investigate a system with large structural changes and naturally denatured protein systems. Although RMA is efficient for a longer simulation than the longest relaxation time of the system, it can also extract rare events in a finite-time simulation such as that conducted at the microsecond scale. By examining the extent to which the correlation function can be reconstructed, we can clarify the information that can be obtained on dynamics using the obtained relaxation modes and rates. Theoretical studies to compare data of the Markov state model with experimental data from nuclear magnetic resonance and neutron scattering analyses have emerged recently (Xia et al. 2013; Lindner et al. 2013; Zheng et al. 2013; Bowman et al. 2014). In the future, it will also be important to interpret the theoretical relationships in light of experimental data.
